# ^1^H-NMR and LC-MS Based Metabolomics Analysis of Wild and Cultivated *Amaranthus* spp.

**DOI:** 10.3390/molecules26040795

**Published:** 2021-02-04

**Authors:** Nolitha Nkobole, Gerhard Prinsloo

**Affiliations:** Department of Agriculture and Animal Health, Science Campus, University of South Africa, Florida 1710, South Africa; prinsg@unisa.ac.za

**Keywords:** phytochemicals, *Amaranthus*, nuclear magnetic resonance (NMR)-based metabolomics, liquid chromatography-mass spectrometry (LC-MS)

## Abstract

*Amaranthus* crops are important for their use as food and nutritional sources, as well as for their medicinal properties. They are mostly harvested from the wild, and cultivation of *Amaranthus* species is still rare, and therefore, attempts are being made to commercialize and market this important crop. This research investigated the effect of cultivation and environment on the chemical profile of both cultivated and wild *A. cruentus* and *A. hybridus* by multivariate statistical analysis of spectral data deduced by Nuclear Magnetic Resonance (NMR). Furthermore, wild samples of *A. cruentus* and *A. hybridus* were subjected to Liquid Chromatography-Mass Spectrometry (LC-MS) for further analysis. Through NMR analysis, it was found that maltose and sucrose increased in both cultivated *A. cruentus* and *A. hybridus*. Moreover, the amino acid, proline was present in cultivated *A. cruentus* in high quantity whereas, proline and leucine were prominent in *A. hybridus*. Other compounds that were found in both wild and cultivated *A. cruentus* and *A. hybridus* are trehalose, trigonelline, lactulose, betaine, valine, alanine, fumarate, formate and kynurenine. LC-MS analysis revealed the presence of rutin, 2-phenylethenamine and amaranthussaponin I in both wild *A. cruentus* and *A. hybridus,* while chlorogenic acid was identified only in cultivated *A. hybridus*. On the contrary, L-tryptophan, kaempferol, phenylalanine and quercetin were detected only in wild *A. cruentus*. Amaranth is not only rich in macro and micronutrients, but the leaves also contain phytochemicals that vary between species and cultivated plants, and might, therefore, affect the medicinal properties of the material.

## 1. Introduction

*Amaranthus* has been rediscovered as a promising food crop mainly due to its resistance to biotic and abiotic factors and the high nutritional value of both seeds and leaves [1]. Amaranth leaves are rich economic sources of carotenoids and proteins, including the essential amino acids methionine and lysine [2]. In addition, the vegetables are packed with dietary fiber and minerals, such as magnesium, calcium, potassium, copper, phosphorus, zinc, iron, and manganese [3]. In South Africa, the leaves are favored to supplement starch-based diets [4].

Edible plants, especially leafy vegetables, also contain phytochemicals, which have potential to impact human health positively. Many studies have reported that Amaranth leaves are a rich source of diverse bioactive compounds such as gallic acid, ferulic acid, and quercetin which contribute to its health promoting attributes [5,6,7]. For an example, α-spinosterol and squalene, isolated from *A. spinosus*, demonstrated antidiabetic, antifungal, and antitumor properties [6,8]. Ferulic acid, which has been isolated from Amaranth, possesses anti-inflammatory, antioxidant, antimicrobial activity, anticancer, and antidiabetic effects [9]. Gallic acid, which is another constituent isolated from Amaranth, has been reported to have many beneficial effects, including antioxidant, anti-inflammatory and antineoplastic properties [10]. *Amarathus* spp. are largely gathered from the wild, even though few selected cultivation incidences have been recorded [11]. Since various compounds in *Amaranthus* leaves have been linked to the plant’s health-promoting effects, it is important to better understand compound profiles depending on the conditions of cultivation and environmental factors.

To date, there has been no documentation of metabolomics profiles linked to geographical origin and the impact of cultivation on *Amaranthus* species. There is a growing interest in using metabolomics technology because it does not require a priori knowledge of the species’ chemical composition [12]. Metabolomics provides insights into the fundamental nature of plant phenotype in relation to development, physiology, and environment [13]. Several analytical methods are applied in metabolomics, however, compared to other methods, proton nuclear magnetic resonance (^1^H-NMR) is widely used [14,15]. This analytical method allows a wide range of metabolites to be identified simultaneously, offering an accurate and easily reproducible representation of the plant’s metabolome [16]. However, compared to Liquid Chromatography-Mass Spectrometry (LC-MS), NMR is deemed less sensitive [17]. Moreover, MS is preferred for its excellent sensitivity, high specificity, and simplicity without elaborate sample preparatory procedures [18]. It can, thus, further enhance the resolution of unknown metabolites, particularly for isomer metabolites and poor chromatographic separation [19].

This study explores the metabolome of wild and cultivated Amaranth leaves using ^1^H-NMR and LC-MS to explain the general variations in chemical composition between wild and cultivated plants, and to assist and guide commercial production of Amaranth.

## 2. Results

### 2.1. Metabolite Composition of A. cruentus Grown in Gauteng and KZN Provinces

**Figure 1:**
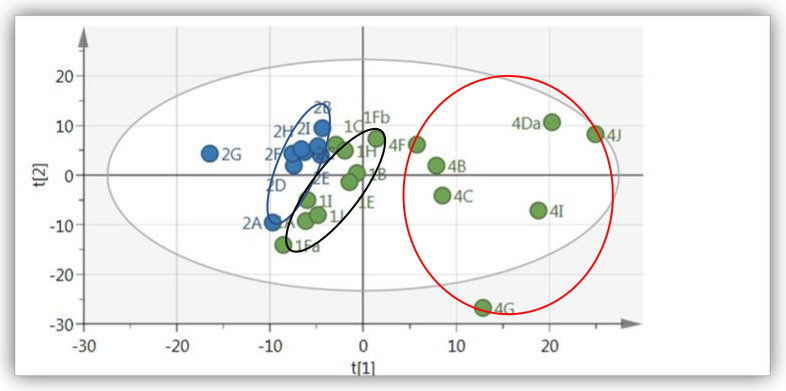
Score scatter plot of PCA of *A. cruentus* leaf extracts (wild and cultivated). Green = cultivated at Mothong in the open field (red circle) and shade net (black circle), blue = collected from the wild (KZN) (R^2^X = 0.95 and Q^2^ = 0.86).

The PCA analysis of ^1^H-NMR spectra of methanol extracts of wild and cultivated (field and shade net) *A. cruentus* is shown in Figure 1. Each point in the PCA scatter plot is a representation of an individual sample. The two first axes Principal component (PC) PC1 and PC2 explained 86.4% of the variance (with PC1 and PC2 describing 72.6% and 13.8% of the variance, respectively). In addition, the model showed a good fit (R^2^X _(cum)_ = 0.95) and predictive ability (Q^2^_(cum)_ = 0.86). Most of the wild samples clustered together (blue) and showed positive loading along PC2 with the exception of one sample. The cultivated *A. cruentus* species, separated into two clusters for the open field (red circle) and shade net (black circle), although the open field samples were distributed throughout the plane.

**Figure 2:**
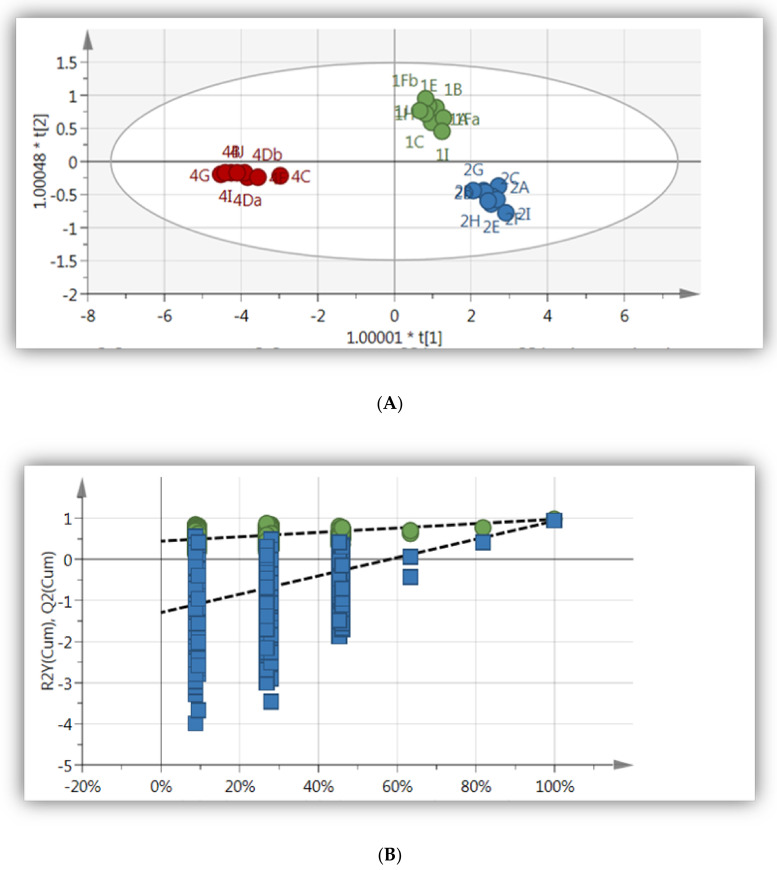

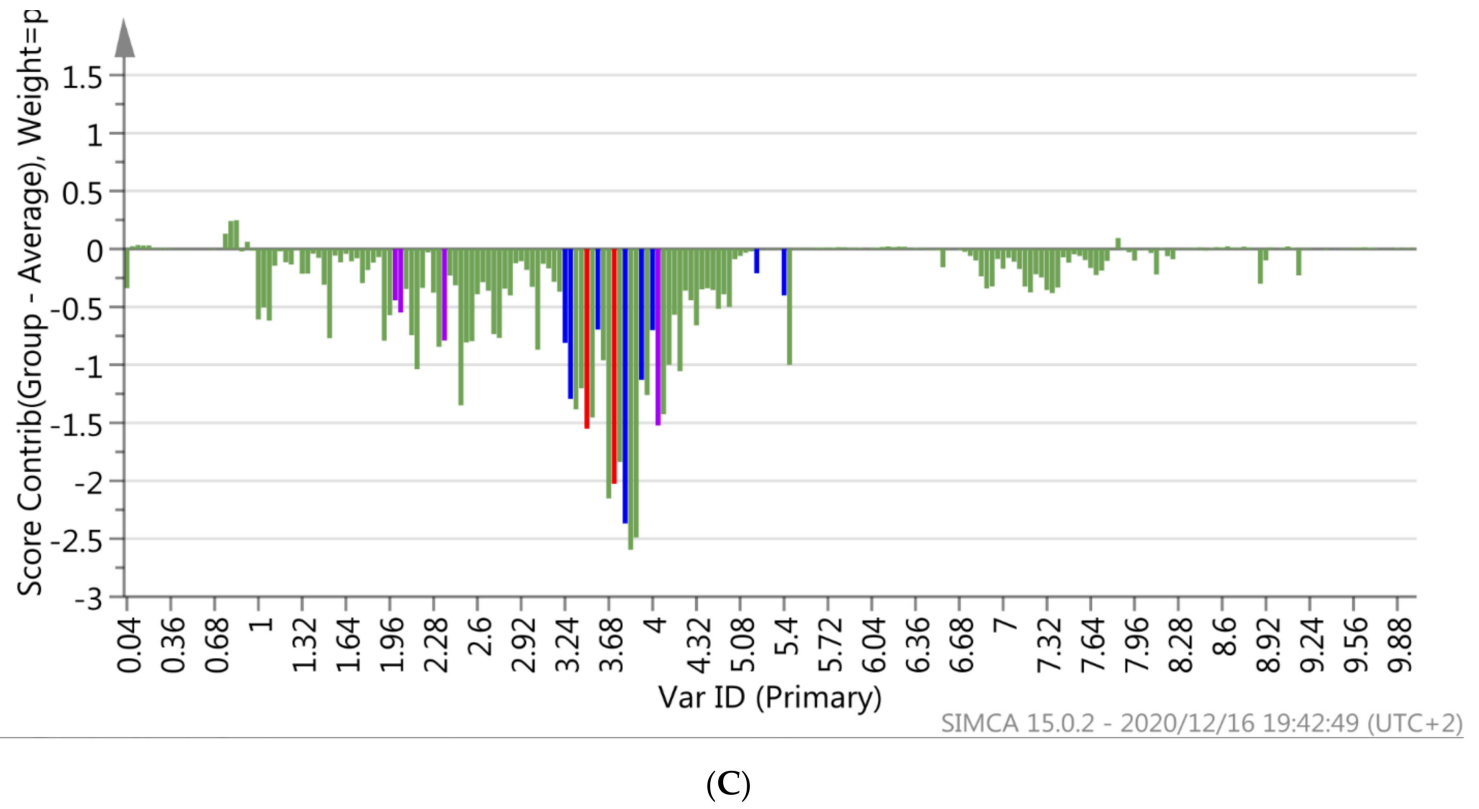
(**A**) Score scatter plot of OPLS-DA of *A. cruentus* leaf extracts (wild and cultivated). Blue = Collected from the wild (KZN), Green = Cultivated at Mothong in the shade net, and Red = Cultivated at Mothong in the open field (R^2^X = 0.95, R^2^Y = 0.89 and Q^2^ = 0.82). (**B**) The response permutation test (*n* = 100) for the OPLS-DA model corresponding to y-axis intercepts (Table): R^2^ = (0.0, 0.44) and Y^2^ = (0.0–1.29). (**C**) Contribution plot generated by comparing wild samples from KZN to cultivated samples (shade net). The blue, red and violet bars represent NMR regions that are associated with maltose, sucrose and proline, respectively. There may be overlaps between NMR regions of maltose, sucrose and maltose.

To improve the clustering and to identify the metabolites responsible for the differences between the samples collected from different areas, an OPLS-DA model (Figure 2A) was constructed. The OPLS-DA statistical model showed a clear distinction between extracts of the cultivated (shade net and open field) and wild *A. cruentus*. The model showed a good fit and predictability as presented by R^2^X = 0.95, R^2^Y = 0.89 and Q^2^ = 0.82 (Table 1, Figure 2), respectively. Moreover, in order to validate the predictive capability of the computed OPLS-DA models, a response permutation test (with *n* = 100) was constructed. This statistical test compares the R^2^ and Q^2^ values of the true model to the permutated model. The test is conducted by assigning the two different groups at random, after which the OPLS-DA models are fitted to each permutated class variable. The values of R^2^ and Q^2^ for the permutated models are then determined and compared with the values of the true models. The results indicate that the measured models have much higher R^2^ and Q^2^ (see Figure 2B) values and, therefore, the OPLS-DA model is validated as it is statistically much better than the 100 permutated model.

To demonstrate the most relevant variables that affected the sample differentiation between the samples, a corresponding contribution plot was produced (Figure 2C). The contribution plots showed that the sugars and aliphatic compounds mostly contributed to the differences between the samples that were cultivated at Mothong (in the shade net) and the wild samples that were collected from KZN. NMR regions of primary and secondary metabolites, responsible for separating wild and cultivated *A. cruentus*, are observed in the contribution plot (Figure 2C) by noting the regions of the chemical shifts. Identified compounds that distinguished cultivated samples from wild samples were maltose, sucrose and proline (Table 2).

### 2.2. Metabolite Composition of A. hybridus Grown in Gauteng and KZN Provinces

**Figure 3:**
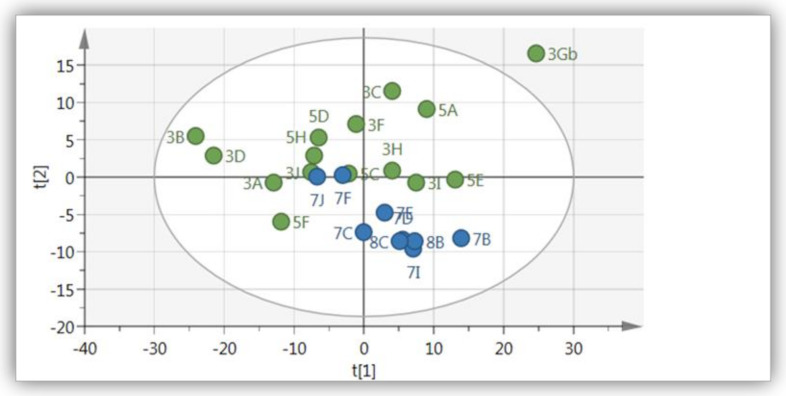
PCA score scatter plot of *A. hybridus* leaf extracts (wild and cultivated). Green = Cultivated at Mothong in the shade net and open field, Blue = Collected from the wild (KZN) (R^2^X = 0.94 and Q^2^ = 0.81).

The PCA analysis of ^1^H-NMR spectra of methanolic water extracts of wild and cultivated *A. hybridus* is illustrated in Figure 3. Principal component (PC) 1 and PC2 explained 78.7% of the variance (with PC1 and PC2 describing 55.8% and 22.9% of the variance, respectively) in the model. Moreover, the model showed a good fit (R^2^X _(cum)_ = 0.94) and predictive ability (Q^2^_(cum)_ = 0.81). With the exception of few samples, a majority of the wild samples grouped together and showed positive loading along PC1 (Figure 3). Cultivated samples showed no order in terms of clustering together as these samples were dispersed throughout the plane.

**Figure 4:**
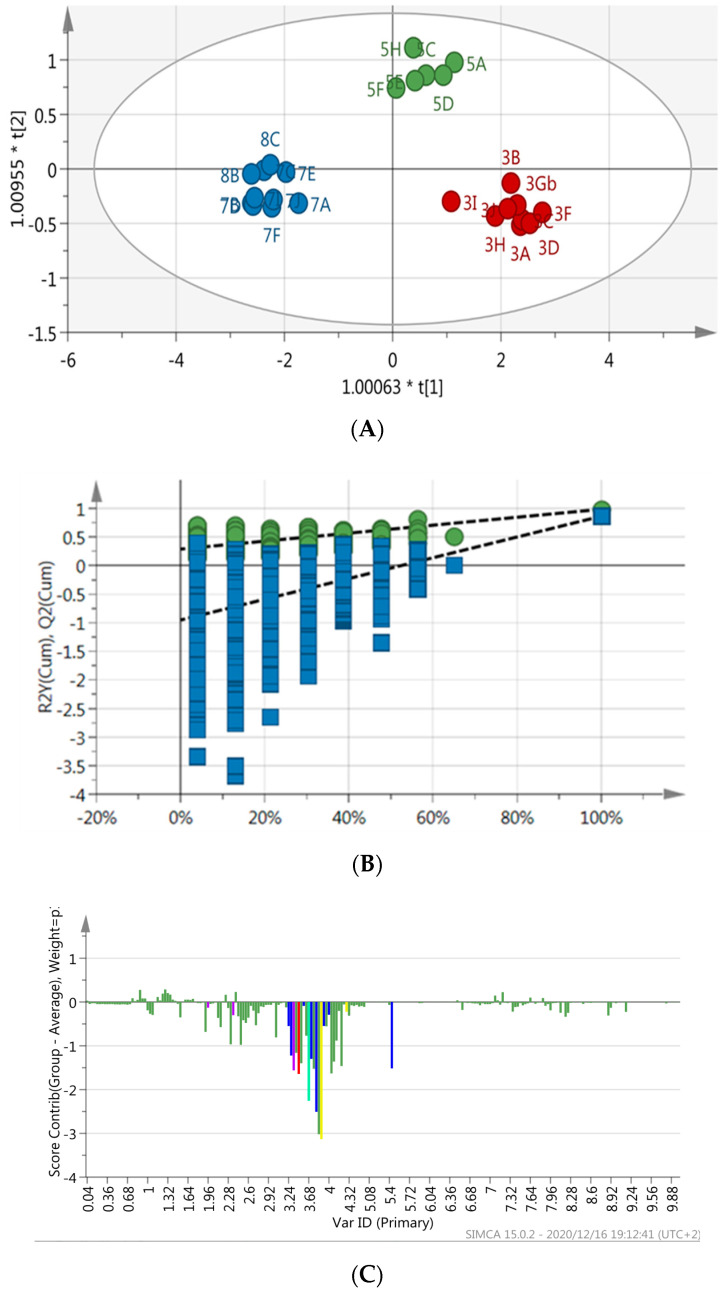
(**A**) OPLS-DA score scatter plot of *A. hybridus* leaf extracts (wild and cultivated). Green = Cultivated at Mothong in the shade net, Red = Cultivated at Mothong on the open field, and Blue = Collected from the wild (KZN) (R^2^X = 0.93, R^2^Y = 0.88 and Q^2^ = 0.82). (**B**) The response permutation test (*n* = 100) for the OPLS-DA model corresponding to y-axis intercepts: R^2^ = (0.0, 0.28) and Y^2^ = (0.0–0.95). (**C**) Contribution plot generated by comparing wild samples from KZN to cultivated samples (shade net). The blue, red and violet bars represent NMR regions that are associated with maltose, sucrose and proline, respectively. In addition, teal and yellow bars represent NMR regions associated with leucine and chlorogenic acid, respectively. There may be overlaps between NMR regions of identified metabolites.

The supervised multivariate analysis model (OPLS-DA) showed a clear discrimination between the sample groups within the 95% confidence interval (Figure 4A). The computed model showed a good fit and predictability as presented by R^2^X = 0.93, R^2^Y = 0.88 and Q^2^ = 0.82 (Table 3, Figure 4A), respectively. Furthermore, the predictive capability of the computed OPLS-DA model was validated using the response permutation test (with *n* = 100). This statistical test compares the R^2^ and Q^2^ values of the true model the permutated model. Once again, it was observed that the true models have much higher R^2^ and Q^2^ (Figure 4B) values as compared with the permutated models.

The contribution plot (Figure 4C) showed that the aliphatic, aromatic and sugar regions were strongly associated with samples that were cultivated at Mothong (shade net) and were negatively associated with the wild samples from KZN. Compounds that distinguished cultivated samples from wild samples were detected as maltose, sucrose, leucine, proline and chlorogenic acid, the latter was only present in the samples grown under shade net (Table 3). Both cultivated and wild samples showed the presence of trigonelline, lactulose, trehalose, betaine, valine, alanine, fumarate, formate, kynurenine and *trans*-4-Hydroxy-L-proline.

The detected metabolites are presented in Table 3. Chenomx and HMDB were used to support the annotation and were compared to published data.

**Figure 5:**
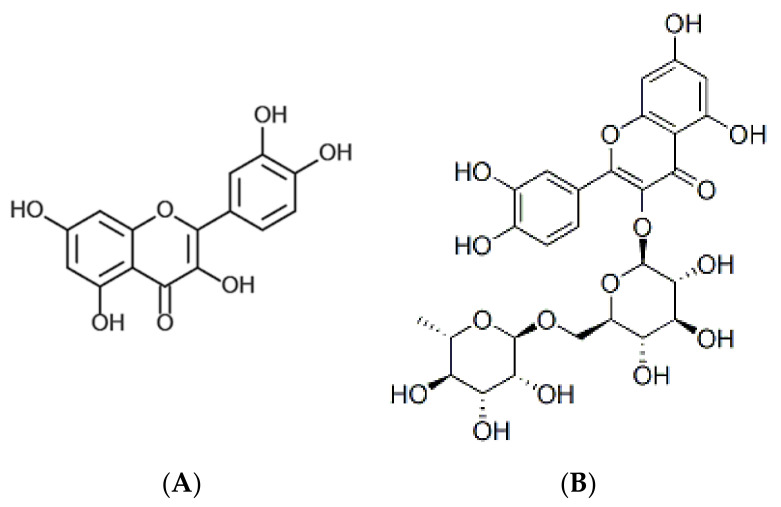
Structures of (**A**) quercetin and (**B**) rutin.

By comparing the NMR regions in the contribution plots that differentiated the wild from the cultivated samples and stacked spectra (Supplementary Materials), the NMR regions indicated the presence of 2-phenylethenamine or a related compound. A list of compounds expected to be present in the samples based on previous studies of other *Amaranthus* spp. was compiled to confirm their presence in the current study. The compounds 2-phenylethenamine and 2-phenylethylamine which were not identified in *Amaranthus* spp. before were also included in the list to confirm their presence. LC-MS analysis tentatively identified nine compounds (Table 4) from wild *A. cruentus*, four of which were also present in *A. hybridus*. Compounds such as quercetin and rutin which were previously identified in *A. caudatus*, were identified in *A. cruentus* for the first time (Table 4, Figure 5). Compounds such as ferulic acid and vanillic acid which were previously identified in Amaranth, were not detected in any of the species in this study.

## 3. Discussion

Amaranth being an important food source and treatment for various diseases, necessitates research on cultivation and how it affects the metabolome of plants. In the present study, two Amaranth species were investigated to determine the effect of geographical location and cultivation. NMR-based metabolomic analysis showed distinct clustering of samples in the OPLS-DA models for the wild, cultivated in the open field, and plants cultivated under shade net. The clustering was observed for both *A. cruentus* and *A. hybridus* and is indicative of a response for both species to the cultivation conditions. To determine which compounds were affected by the geographical as well as cultivation conditions, contribution plots and databases such as Chenomx and HMDB were used for annotation. The sugar regions for both species (Figure 2 and Figure 4) showed an increase in sugar concentration in the region 3.4–4.0 ppm for cultivated material. Maltose and sucrose were identified and were more prominent in cultivated *A. cruentus* and *A. hybridus* than in wild species, even though more pronounced for *A. cruentus* (Figure 2C), than for *A. hybridus* (Figure 4C), indicating a difference between the species. The low concentration of maltose in wild *A. cruentus* and *A. hybridus* suggests that the conditions for the wild grown material caused the soluble sugar concentration to decrease in the wild plants compared to the cultivated samples. The more favorable conditions under cultivation such as addition of fertilizer and watering (even though it was limited), might also support general better growth and development, hence the increase in soluble sugars in the cultivated material. Even though wild grown material was probably exposed to more harsh conditions than the cultivated material, there were also distinct clustering for the cultivated material grown in the open field when compared to the plants grown under shade net (Figure 2A and Figure 4A). This clustering is, therefore, indicative of the effect of shade on the chemical profile, as the amount of watering and soil nutrients were the same.

Aside from sugars, amino acids were also differentiated in the samples. Plants accumulate an array of metabolites, particularly amino acids, when exposed to stressful conditions. A report by Hayat et al. (2012) [27] suggests a positive correlation between proline accumulation and plant stress. Proline, an amino acid, plays a crucial role in plants exposed to various stress conditions. Moreover, some studies suggest that proline not only accumulates during abiotic stress, but also in different tissues of plants under non-stress conditions [28] playing a role in plant growth and development. In line with these finding by Hayat and colleagues (2012) [27], this study deduces that the accumulation of proline in cultivated samples (*A. cruentus* and *A. hybridus*) could have been influenced by the environmental factors at play since both plants were cultivated and collected in the same area. Other explanation for the accumulation of high proline in cultivated species could linked to the plant’s development state at time of collection. Branched chain amino acid (BCAAs) such as leucine and valine were also increased in the cultivated material. Leucine was detected in cultivated *A. hybridus*, whereas valine was found in cultivated *A. cruentus*. Bowne et al. (2012) [29] suggested that the increased levels of branched chain amino acids observed in wheat cultivars was attributed to drought experienced by the plants. These results were comparable to the findings of [30] which reported that accumulation of BCAAs, along with a number of other amino acids, increased under the stress of dehydration and was regulated at transcriptional levels. In this study, the results of the previous studies are contradicted, as the two amino acids were differentiated in the two species, and not as a result of water availability. The difference in compounds was also demonstrated by the LC-MS results, where more compounds were present in *A. cruentus* when compared to *A. hybridus* (Table 4). Leucine-rich diet is associated with promoting acute secretion of insulin from pancreatic β cells, hence consumption of leucine-rich foods may aid diabetic patients [31]. In another study, it was established that leucine supplementation improves glucose homeostasis [32], supporting the use of Amaranth for treating diabetes.

Other compounds that were observed in Amaranth include trehalose, chlorogenic acid and trigonelline. Trehalose is a predominantly found non-reducing sugar in bacteria, fungi, yeast, insects, and plants [33]. Significant trehalose levels in plants act as a protectant against various abiotic stresses such as heat, drought, high salinity and ultraviolet rays [34]. The study proposes that the presence of trehalose in all the samples studied is indicative of the ability of Amaranth to endure harsh environmental and climatic conditions. In herbaceous plants, trigonelline is ubiquitous in saline-dried environments [35]. Previously, trigonelline has been identified from young aerial parts of *A. hybridus* [36]. In this study, trigonelline was present in both *A. hybridus* and *A. cruentus* of wild and cultivated origins. Zhou et al. (2013) [37] reported that trigonelline has beneficial effects for diabetes by lowering blood glucose and lipid levels, improved insulin sensitivity index and insulin content. In another study, trigonelline decreased the ratio of kidney weight/body weight and blood glucose levels and lowered blood urea levels of nitrogen, creatinine and albumin for type 2 induced diabetic rats [38]. In the current study, chlorogenic acid was only present in cultivated *A. hybridus*. Chlorogenic acid is one of the most abundant polyphenol compounds in the human diet, belonging to secondary phenolic metabolites produced by certain plant species [39] and was previously identified by HPLC in the leaves of *A. tricolor* [40,41]. Chlorogenic acids contain many health benefits, including antioxidant, chemopreventive and other activities [42] such as lowering the risk of type 2 diabetes mellitus [43]. Kaempferol is a polyphenol antioxidant that is present in fruits and vegetables and has been linked to reducing the risk of chronic diseases, especially cancer [44]. Previous studies showed that kaempferol has been isolated from *A. spinosus* [45]. Quercetin was previously isolated from *A. spinosus* [45]. The LC-MS analysis confirmed the presence of well-known compounds in *Amaranthus* such as kaempferol, quercetin amaranthussaponin I and rutin (Table 4). Trigonelline, also widely reported in Amaranth, is reported for the first time in *A. cruentus* and *A. hydridus* in this study, while 2-phenylethenamine is reported for the first time in *Amaranthus.*

## 4. Materials and Methods

### 4.1. Collection of Wild Vegetables from KwaZulu-Natal (KZN) Province, South Africa

*Amaranthus hybridus* leaves were bought from Esikhawini informal market in April 2018. Esikhawini is a peri-urban settlement situated in the district of Richards Bay in KwaZulu-Natal, and characterized by 1087 mm of rain per year, with most rainfall occurring mainly during summer. Esikhawini receives the lowest rainfall in June (42 mm) and the highest in March (133 mm) (Esikhawini climate, Map of South Africa, ND).

The leaves of *A. cruentus* were purchased from Stanger, which is situated in KwaDukuza in KwaZulu-Natal. Stanger normally receives about 866 mm of rain per year, most of which occurs during the summer (Stanger climate, Map of South Africa, ND). The leaves were sold in pierced plastic bags to avoid sweating of the leaves and to allow some airflow. Upon purchase, the leaves were transferred to brown paper bags to prepare for transportation to the lab. Upon arrival, the leaves were washed and left on the countertop to dry. After drying, they were grounded to a fine powder and stored at room temperature until analysis.

Wild Amaranth plants collected from KwaZulu-Natal were confirmed by a botanist at the University of Zululand, Prof. Alfred Zobolo.

### 4.2. Planting and Harvesting of A. cruentus and A. hybridus

The seeds of *A. cruentus* and *A. hybridus* were donated by the gene bank of the Agriculture Research Council-Vegetable and Ornamental Plants (ARC-VOP) where they are maintained to ensure that the material used in the study was true-to-type. *Amaranthus cruentus* and *A. hybridus* were grown in November 2017 at Mothong African Heritage Centre garden in Mamelodi, Pretoria (GPS co-ordinates: 25°41′49.7″ S 28°20′17.4″ E). Prior to planting in the field and in the shade net, the seeds were sown in 98 cavity seedling trays using hygromix as a growth medium and kept under a 40% shade net. Seedlings were transplanted to the field or in a 40% shade net, 21 days after emergence [46]. The seedlings were planted at a recommended spacing of 10 cm × 20 cm (50 plants m^−2^) in the field and in the shade net. Limestone ammonium nitrate (LAN) at 50 kg N ha^−1^ (containing 28% nitrogen, calcium and magnesium) was applied on the freshly prepared soil on the day of planting the seedlings. Plants were irrigated once a week by hand, as water was limited at the site.

The first harvest was done two months after sowing early in the morning before 10:00 am. For each species, three separate batches were randomly harvested from different positions in the field to obtain a representative sample batch for each species. Each plant was sprinkled with water and each batch loosely packed in a marked and pierced black plastic bag to avoid sweating of the leaves and to encourage airflow. The bags were transported to the laboratory for drying at room temperature. When the leaves were dry, they were ground to a fine powder and stored at room temperature until analysis.

### 4.3. Metabolites Analysis

#### 4.3.1. NMR Spectroscopy

The untargeted metabolomics analysis extraction employed in this study is described in [47,48]. For extraction and analysis, pulverized leaf content of 50 mg per sample was weighed into 2 mL Eppendorf tubes. The samples were suspended in 0.75 mL deuterated methanol (CD_3_OD) and 0.75 mL KH_2_PO_4_, buffered in deuterium water (D_2_O) (pH 6.0) containing 0.01% (*w*/*w*) TSP. The sample mix was vortexed for one minute at room temperature to reach a homogenous state and ultra-sonicated to break down the cell walls for 20 min (Kim et al. 2010^a^). The sample was centrifuged at 13,000 rpm for an additional 20 min to remove the supernatant from the pellet. The supernatant was then transferred to a 5 mm NMR tube for analysis where 32 scans were performed on a 600 MHz NMR spectrometer (Varian Inc., Palo Alto, CA, USA).

NMR spectral data were processed using MestReNova software (9.0.1, Mestrelab Research, Spain) where NMR spectral data were subjected to phase correction, baseline correction, referencing and normalization, after which the spectral intensities were reduced to equal width (0.04 ppm each) integrated regions, corresponding to the region of 0.04–10.00 ppm. The residual water peak (4.70–4.90 ppm) and methanol peaks (3.30–3.36 ppm) were excluded from the final data for further analysis [46]. The MestReNova data files were exported to Microsoft Excel for multivariate data analysis using SIMCA-P software (15.0, Umetrics, Malmo, Sweden). The data were first analyzed using an unsupervised method, principal component analysis (PCA), followed by a supervised model, and orthogonal partial least square discriminatory analysis (OPLS-DA). A scores plot and a contribution plot were used to evaluate sources of variance, and the NMR values from the plots were then used in conjunction with existing databases and published literature for annotation. The permutation test with 100 permutations was performed for validation of the OPLS-DA model.

#### 4.3.2. NMR Data

The important chemical shifts from the untargeted NMR-based metabolomics analysis, identified from the contribution plots, were compared to the chemical shifts of compounds in databases such as Chenomx (Version 8.3) and the Human Metabolome Database. Candidate structures were compared to previously published literature.

#### 4.3.3. Liquid Chromatography-Mass Spectroscopy (LC-MS)

Triple quad-based LC-MS (LC-QqQ-MS) was used in order to confirm the presence of identified compounds in *Amaranthus* spp. and to further detect compounds that could not be identified with NMR metabolomic analysis. A list of compounds previously identified in Amaranth was compiled to guide identification of the compounds in *A. cruentus* and *A. hybridus*. Fifty milligrams of dried *Amaranthus* leaf materials were extracted with 1.5 mL (75% MeOH: 25%) water. Samples were sonicated for five minutes in an ultrasonic bath and further centrifuged for an additional 15 min with the centrifuge table set at 15,000 rpm. To extract and remove cell debris, the supernatant was filtered through Sartorius Minisart RC 4 0.2-micron syringe filters with a 1-mL plastic pipette.

#### 4.3.4. UPLC Analysis

To produce accurate mass data, a Waters UPLC coupled in tandem to a Waters SYNAPT G1 HDMS mass spectrometer was used. The chromatographic separation optimization was performed using a column of Waters HSS T3 C18 (150 mm × 2.1 mm, 1.8 μm) and a column temperature regulated at 60 °C. A binary solvent mixture consisting of water (Eluent A) containing 10 mM formic acid (natural pH of 2.3) and acetonitrile (Eluent B) containing 10 mM formic acid was used. The initial conditions were 98% A at a flow rate of 0.4 mL/min and were maintained for 1 min, followed by a linear gradient to 5% A at 25 min. The conditions were kept constant for 2 min and then changed to the initial conditions. The runtime was 30 min, and the injection volume was 2 µL. Samples were kept cool at 6 °C in the Sample Manager during the analysis. A strong (50% acetonitrile: 50% methanol) and weak (10% methanol in water) needle wash solution was used to eliminate any carry-over between injections.

During the optimization of the chromatographic method each change in solvent composition was evaluated by triplicate injections. This was only done once the system pressure stabilized within a 20 psi pressure ripple (0.2% variance). The optimized method was evaluated for stability and it was found that retention times differed by +/− 0.02 s during triplicate analysis. This stability and flow accuracy were observed in both ionization modes.

#### 4.3.5. TOF MS Analysis

The SYNAPT G1 mass spectrometer was used in V-optics and operated in the electrospray mode to enable detection of phenolic and other ESI-compatible compounds. The system was calibrated for the mass range 50–1500 Dalton using a 100 pg/mL formic acid solution. Leucine enkephalin (50 pg/mL) was used as a reference calibrant to obtain typical mass accuracies between 1 and 5 mDalton (mDa). The lockmass was sampled every 20 s for 0.5 s. The mass spectrometer was operated in both ESI positive and negative modes with a capillary voltage of 2.5 kV, the sampling cone at 30 V, and the extraction cone at 4.0 V. The scan time was 0.1 s covering the 50 to 1200 Dalton mass range. The interscan time was 0.02 s. The detector voltage was 1800 V that is well within the acceptable range. The source temperature was 120 °C, and the desolvation temperature was set at 450 °C. Nitrogen was used as the nebulization gas at a flow rate of 550 L/h, and cone gas was added at 50 L/h. The software used to control the hyphenated system and do all data manipulation was MassLynx 4.1 (SCN 872).

The raw data were processed and extracted ion chromatograms (XICs) obtained based on the known compounds found in published literature. The accurate mass data of each detected compound were submitted for elemental composition, double-bond equivalence (DBE) as well as isotopic fit calculations. Compounds tentatively identified using the above-mentioned criteria are listed in Table 4. Compound identification was further enhanced by analyzing all samples with low- and high- collision energy settings of the collision cell. To minimize compound fragmentation, a low-energy setting of 3 eV was used, but to enhance fragmentation of molecules, various collision energies between 10 and 40 eV were used (MS^E^). Fragmentation spectra were submitted to the NIST mass spectral library (NIST 2014, Version 2.2) as well as the mass spectral libraries developed on the Synapt G1 system.

## 5. Conclusions

LC-MS and NMR technologies were successfully used to tentatively identify compounds from wild and cultivated *Amaranthus* spp. namely, *A. cruentus* and *A. hybridus* collected from different areas. Cultivated samples of *A. cruentus* had higher quantities of maltose, sucrose, and proline than the wild samples. With regard to *A. hybridus*, cultivated samples accumulated more maltose, sucrose, proline, leucine and chlorogenic acid, compared with the quantities which were much lower in wild species. Chlorogenic acid was only found in cultivated samples of *A. hybridus*. Through LC-MS, well-known constituents of *Amaranthus* such as kaempferol and quercetin were confirmed in wild *A. cruentus*, while 2-phenylethenamine is reported for the first time in both *A. cruentus* and *A. hybridus*. The findings in this study demonstrate that different species of *Amaranthus* are responding differently to geographical and cultivation factors, by showing different chemical composition, and subsequently will result in vast and diverse medicinal properties due to chemicals they possess. Using LC-MS and NMR-based metabolomics, it is possible to screen the chemical profile of *Amaranthus* spp. collected from different areas to guide production of *Amaranthus* for commercial purposes.

## Figures and Tables

**Table 1:** Model quality and description of OPLS-DA for *A. cruentus.*

OPLS-DA			Permutation (*n* = 100)	
**R^2^X**	R**^2^Y**	Q**^2^**	R**^2^**	Y**^2^**
0.95	0.89	0.82	(0.0, 0.44)	(0.0–1.29)

**Table 2:** NMR peaks (ppm) of the compounds that contributed to the separation of cultivated (open field) and wild leaf extracts of *A. cruentus* and *A. hybridus.*

Group	Compound	H-NMR Chemical Shift (ppm)	Chenomx (ppm)	Human Metabolite Database	Reference (ppm)	Literature
Cultivated (Shade Net)	Maltose	3.24	3.3	3.27		[20]
		3.4	3.4	3.41		
		3.6	3.6	3.66		
		3.72	3.7	3.70		
		3.8	3.8	3.84		
		3.92	3.9	3.9		
		4.0	4.0	3.96		
		4.56	4.6			
		5.2	5.2	5.22	5.34	
		5.4	5.4	5.40	5.42	
	Sucrose	3.52	3.5	3.46	3.53	[21]
		3.6	3.6	3.55	3.67	
		3.72	3.7	3.75		
		3.8	3.8	3.82	3.87	
		3.92	3.9	3.89		
		4	4.0	4.04	4.07	
		4.2	4.2	4.21	4.16	
		5.41	5.4	5.4	5.39	
	Proline	1.99	2.0	1.96	1.98	[20]
		2.06	2.0	2.04	2.02	
		2.34	2.3	2.32		
		3.33	3.3	3.24		
		3.41	3.4	3.4		
		4.1	4.1			
	* Leucine	0.88	0.9	0.94	0.94	[20]
		1.0	1.0			
		1.68	1.7	1.70	1.70	
		3.68	3.7			
	** Chlorogenic acid	2.0	2.0	2.02	2.03	[22]
		2.08	2.1	2.17		
		2.2	2.2			
		3.88	3.9	3.88	3.80	
		4.28	4.3	4.25	4.23	
		5.28	5.3	5.33	5.33	
		6.4	6.4	6.39	6.39	
		6.88	6.9	6.94		
		7.08	7.1	7.12	7.05	
		7.2	7.2	7.19		
		7.6	**7.6**	7.65	7.59	
Wild and Cultivated	Trigonelline	4.4	4.4	4.42	4.41	[21,23]
		8.12	8.1	8.07	8.07	
		8.8	8.8	8.82	8.83	
		9.12	9.1	9.11	9.14	
	Trans-4-Hydroxy-l-proline	2.4	2.4		2.6	[24]
		3.4	3.4			
		3.48	3.5		3.63	
		4.28	4.3			
		4.56	4.7			
	Lactulose	3.6	3.6	3.582	3.5	[25]
		3.68	3.7	3.732	3.6	
		3.8	3.8	3.836	3.8	
		3.88	3.9	3.919	3.9	
		4	4.0	4.012	4.0	
		4.08	4.1	4.125	4.1	
		4.12	4.2	4.2	4.2	
		4.28	4.3	4.249	4.3	
		4.4	4.4	4.286	4.4	
		4.48	4.5	4.46	4.5	
		4.56	4.6	4.553	4.6	
	Trehalose	3.41	3.4	3.44		[20]
		3.6	3.6	3.64		
		3.8	3.8	3.76		
		3.92	3.9	3.88		
		5.2	5.2		5.18	
	Betaine	3.24	3.3	3.25	3.22	[20]
		3.92	3.9	3.89	3.86	
	Valine	1.0	1.0	1.02	1.05	[20,21]
		2.32	2.3	2.26	2.22	
		3.6	3.6	3.60	3.60	
	Alanine	1.44	1.5	1.47	1.47	[26]
		3.8	3.8	3.77		
	Fumarate	6.52	6.5	6.51	6.5	[20]
	Formate	4	8.4	8.44	8.46	[20]
	Kynurenine	3.72	3.7	3.67	3.71	[26]
		4.12	4.1	4.10	4.15	
		6.8	6.8	6.75	6.80	
		6.92	6.9	6.83		
			7.4	7.37	7.44	
		7.8	7.8	7.79	7.84	

* High only in *A. hybridus*. ** detected only in *A. hybridus.*

**Table 3:** Model quality and description of OPLS-DA for *A. hybridus.*

OPLS-DA			Permutation (*n* = 100)	
**R^2^X**	**R^2^Y**	**Q^2^**	**R^2^**	**Y^2^**
0.93	0.88	0.82	(0.0, 0.44)	(0.0–0.95)

**Table 4:** Compounds identified from the wild *Amaranthus hybridus* and *A. cruentus* extracts analyzed by LC-QqQ-MS.

Compound	Empirical Formula	Ret time (Min)	Measured Mass	Theoretical Mass	Mass Error (mDa)	DBE	Mode (+/−)	*Amaranthus Cruentus*	*Amaranthus Hybridus*
2-Phenylethenamine	C_8_H_9_N	1.72	1,200,809	1,190,735	0.4	5	+	✓	Trace
2-Phenylethylamine	C_8_H_11_N	-	-	1,210,891	-	-	ND	✗	✗
l-Tryptophan	C_11_H_12_N_2_O_2_	2.62	2,050,977	2,040,899	1.4	7	+	✓	✗
Phenylalanine	C_9_H_11_NO_2_	1.85	1,660,851	16,507,890	1.7	5	+	✓	✗
Kaempferol	C_15_H_10_O_6_	7.22	2,870,559	2,860,477	0.3	11	+	✓	✗
Ferulic acid	C_10_H_10_O_4_	-	-	1,940,579	-	-	+	✗	✗
Epicatechin	C_15_H_14_O_6_	-	-	2,900,790	-	-	ND	✗	✗
Chlorogenic acid	C_16_H_18_O_9_	-	-	3,540,951	-	-	ND	✗	✗
Vanillic acid	C_8_H_8_O_4_	-	-	1,680,422	-	-	ND	✗	✗
Quercetin	C_15_H_10_O_7_	6.66	3,030,477	3,022,357	0.2	-	+	✓	✗
Rutin	C_27_H_30_O_16_	6.41	6,091,456	6,101,534	1.9	13	+/−	✓	✓
Trigonelline	C_7_H_7_NO_2_	0.93	1,380,587	1,370,477	3.2	5	+	✓	✓
Amaranthussaponin I	C_48_H_76_O_19_	15.51	9,554,903	9,564,981	2.6	11	-	✓	✓

✓ Compound present in the sample. ✗ Compound absent in the sample.

## Data Availability

The datasets used and/or analyzed during the current study are available from the corresponding author on reasonable request.

## Supplementary Materials

The following are available online at. Figure S1: The 600 MHz ^1^H-NMR spectra of *A. cruentus* leaf extracts (wild and cultivated). Blue = Cultivated at Mothong in the open field, Green = Collected from the wild (KZN), and Red = Cultivated at Mothong in the shade net, Figure S2: The 600 MHz ^1^H-NMR spectra of *A. hybridus* leaf extracts (wild and cultivated). Blue = Collected from the wild (KZN), Green = Cultivated at Mothong in the shade net, and Red = Cultivated at Mothong on the open field, Figure S3: Comparison of wild (blue spectrum) and cultivated *A. hybridus* (red spectrum). Peaks at δ 5.75, 7.07, 7.29, 7.34 and 7.52 are indicated by arrows. Height of the peaks are indicative of the concentration of the compound, Figure S4: Comparison of wild (red spectrum) and cultivated *A. cruentus* (blue spectrum). Peaks at δ 5.75, 7.10, 7.29, 7.39 and 7.50 are indicated by arrows. Heights of the peaks are indicative of the concentration of the compound.

## Sample Availability

Plant samples excluding compounds are available upon request.
