# Supplementary material for: 1H-NMR and LC-MS Based Metabolomics Analysis of Wild and Cultivated Amaranthus spp
Source: Molecules. 2021 Feb 4;26(4):795. doi: 10.3390/molecules26040795 (PMC7913636; doi:10.3390/molecules26040795)
Supplement: Supplementary file 1 [file molecules-26-00795-s001.pdf]

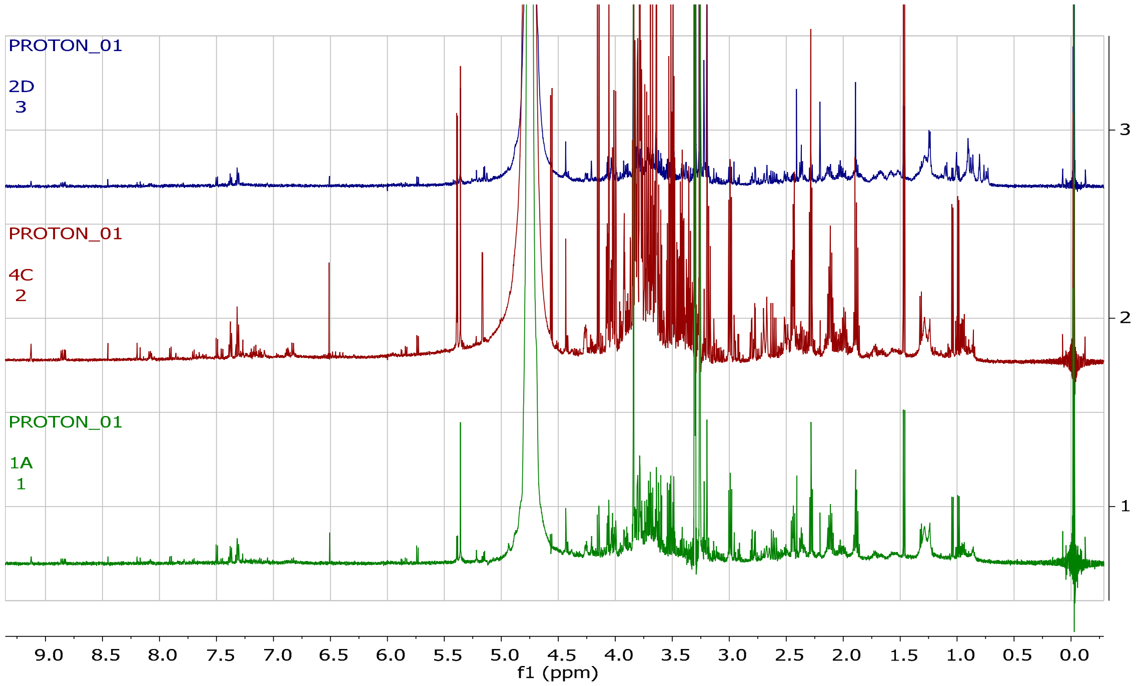

Figure S1. The 600 MHz ¹H-NMR spectra of *A. cruentus* leaf extracts (wild and cultivated). Red = cultivated at Mothong in the open field, blue = collected from the wild (KZN), and green = cultivated at Mothong in the shade net.

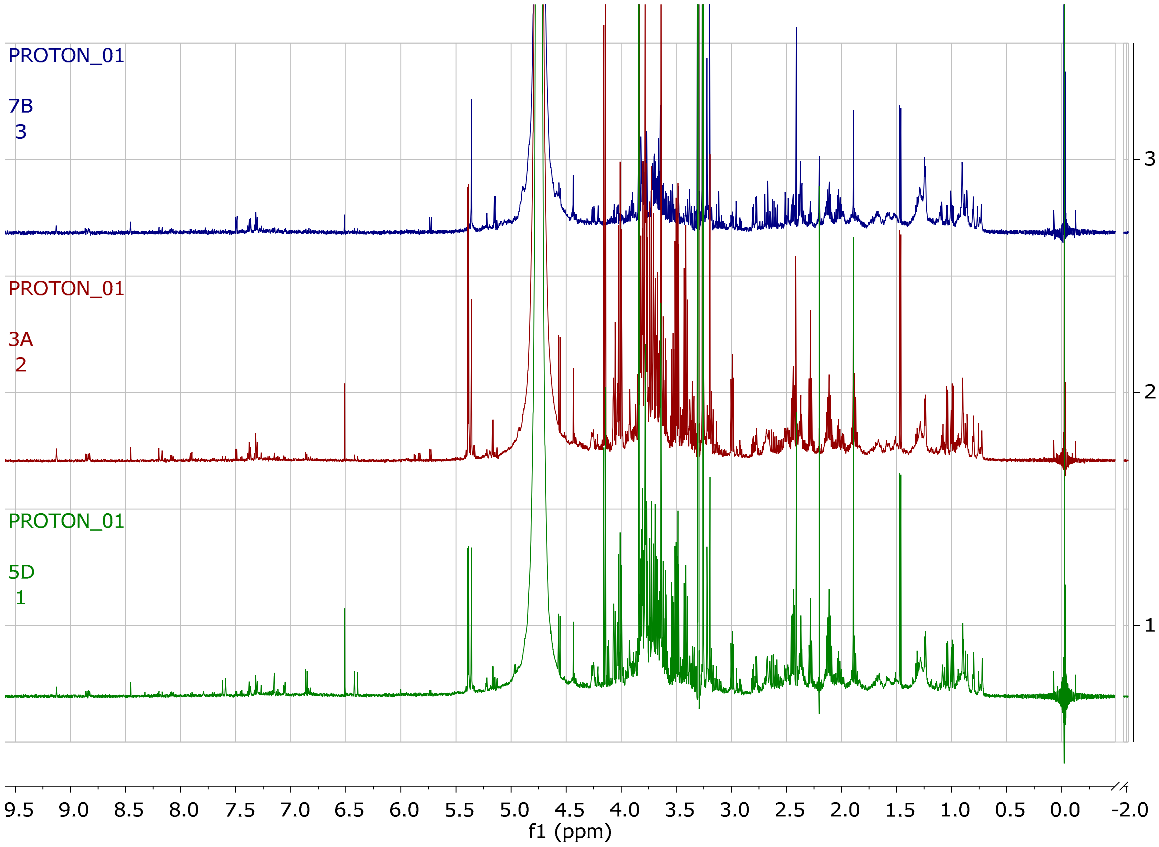

Figure S2. The 600 MHz ¹H-NMR spectra of *A. hybridus* leaf extracts (wild and cultivated). Red = cultivated at Mothong in the open field, blue = collected from the wild (KZN), and green = cultivated at Mothong in the shade net.

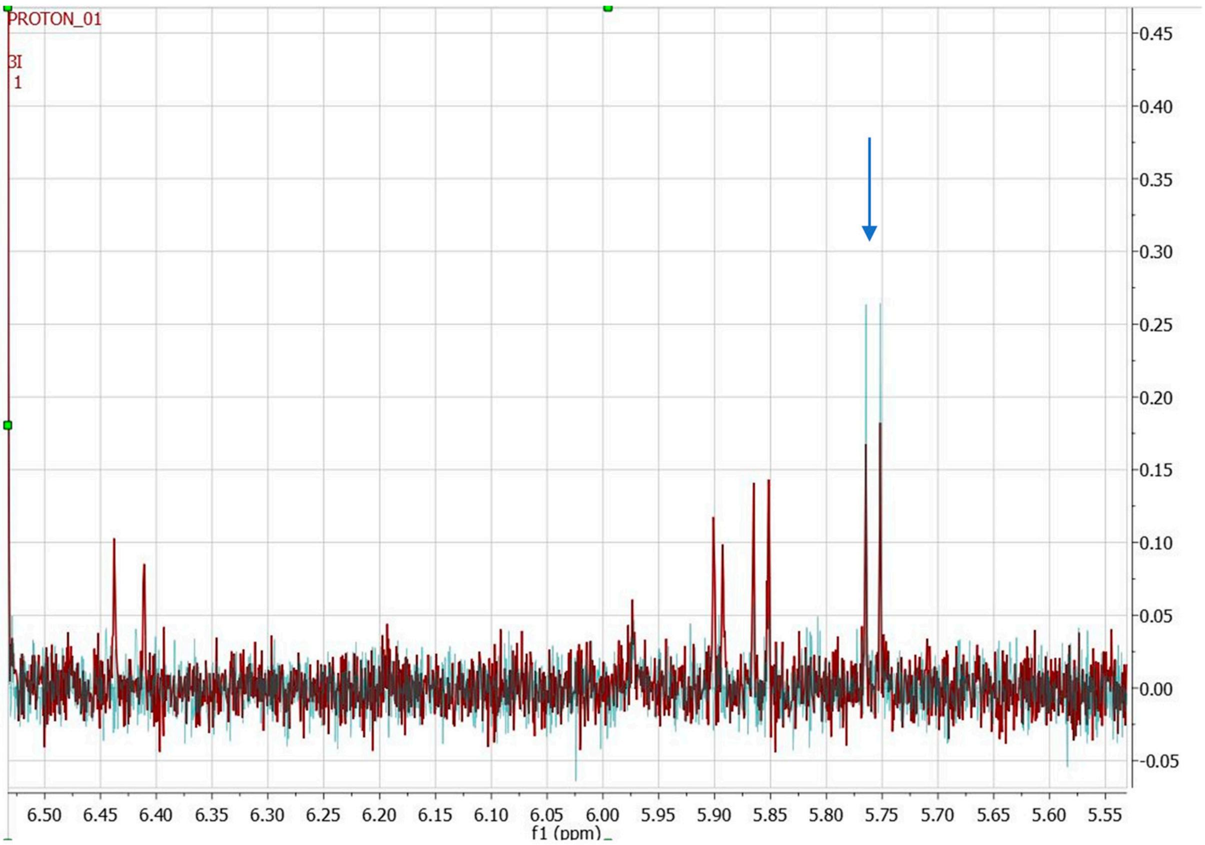

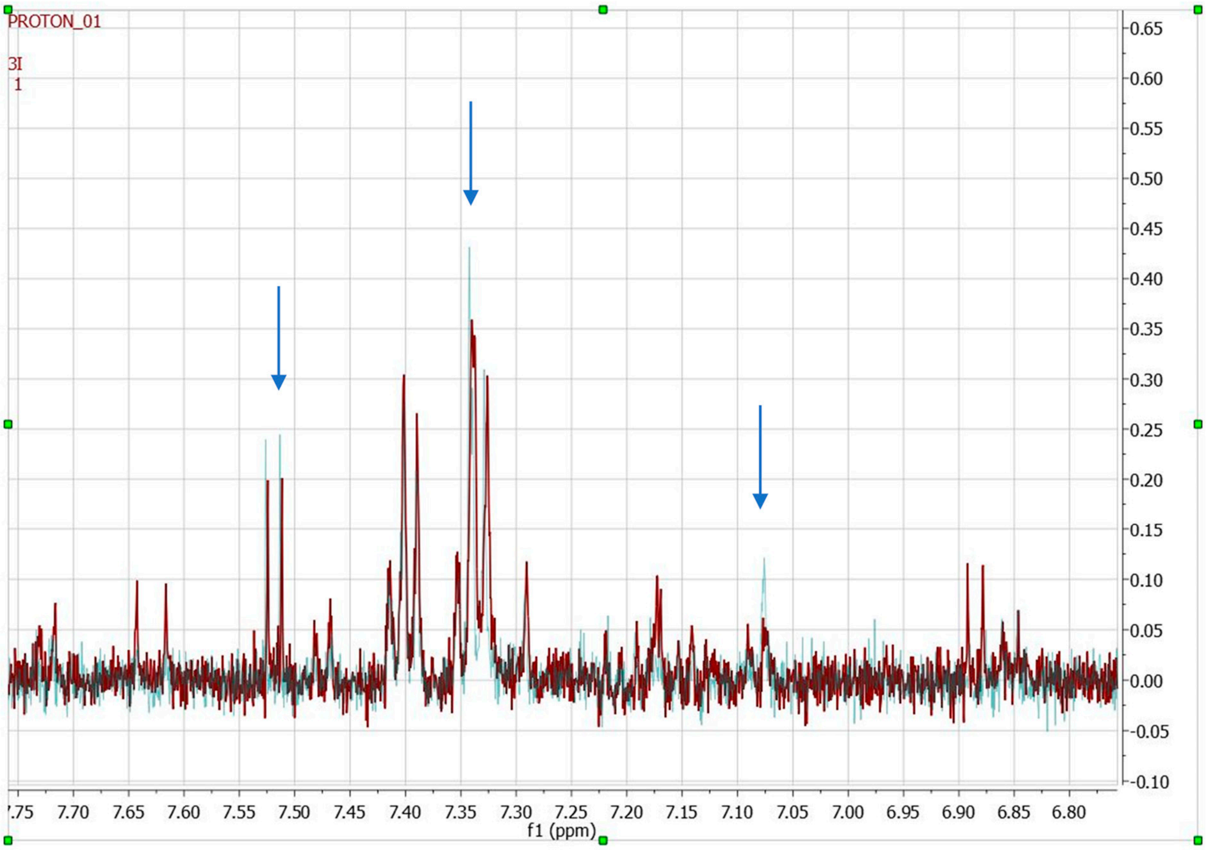

Figure S3. Comparison of wild (blue spectrum) and cultivated *A. hybridus* (red spectrum). Peaks at δ 5.75, 7.07, 7.29, 7.34 and 7.52 are indicated by arrows. Height of the peaks are indicative of the concentration of the compound.

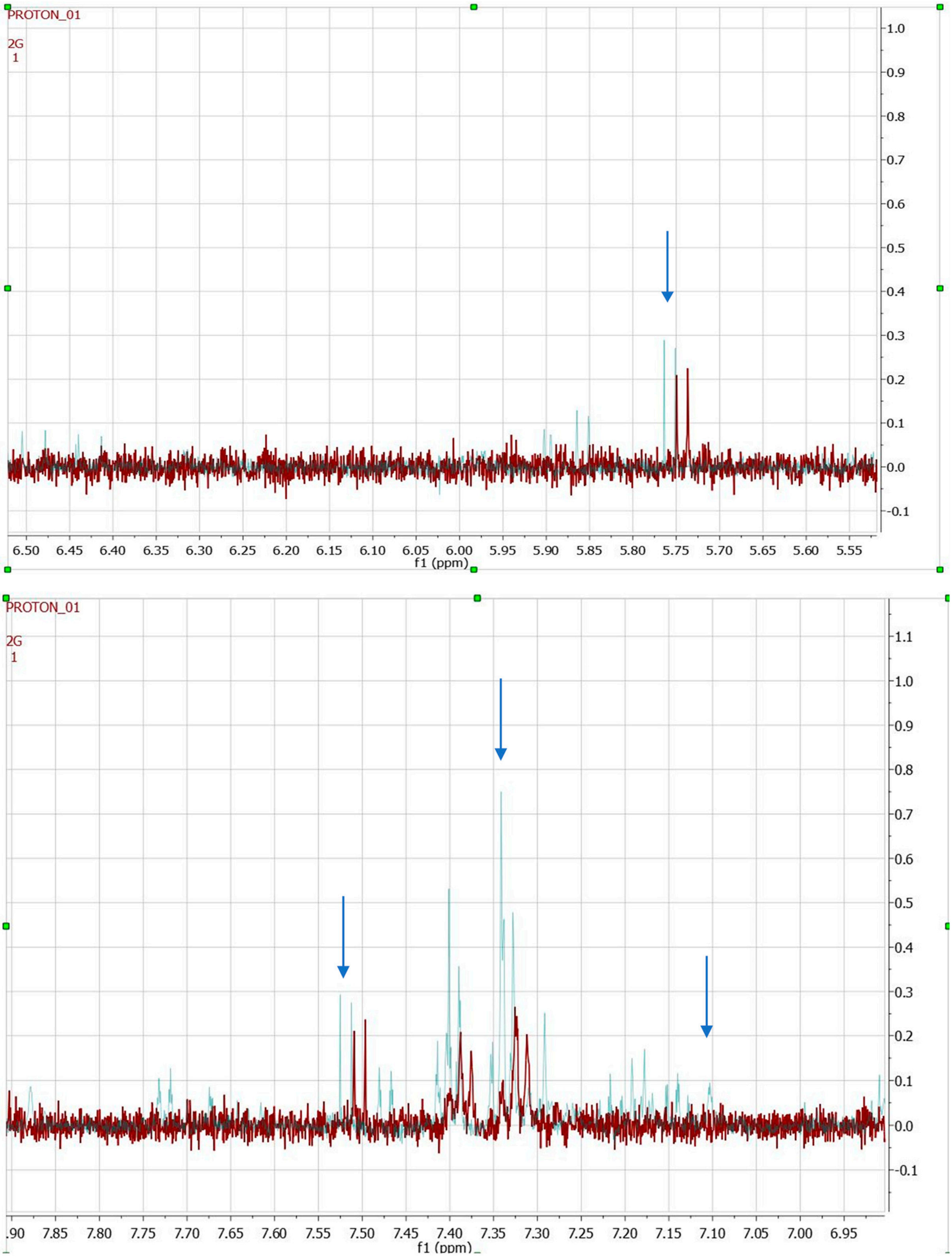

Figure S4. Comparison of wild (red spectrum) and cultivated *A. cruentus* (blue spectrum). Peaks at δ 5.75, 7.10, 7.29, 7.39 and 7.50 are indicated by arrows. Height of the peaks are indicative of the concentration of the compound.
